# Chemical Characterization and Biological Activities of Essential Oil Obtained from Mint Timija Cultivated under Mineral and Biological Fertilizers

**DOI:** 10.1155/2017/6354532

**Published:** 2017-11-12

**Authors:** Ayoub Kasrati, Chaima Alaoui Jamali, Robert Spooner-Hart, Laurent Legendre, David Leach, Abdelaziz Abbad

**Affiliations:** ^1^Faculté des Sciences Semlalia, Université Cadi Ayyad, Marrakech, Morocco; ^2^Ecole Normale Supérieure de Tétouan, Université Abdelmalek Essaadi, Tétouan, Morocco; ^3^School of Science and Health, Western Sydney University, Locked Bag 1797, Penrith, NSW 2751, Australia; ^4^Université de Lyon, 69622 Lyon, France; ^5^Université Lyon 1, Villeurbanne, France; ^6^CNRS, UMR5557, Ecologie Microbienne, Villeurbanne, France; ^7^INRA, UMR1418, Villeurbanne, France

## Abstract

Cultivation of mint timija (*Mentha suaveolens* subsp.* timija* (Briq.) Harley) constitutes a promising solution to the conservation and sustainable utilization of this Moroccan endemic and threatened species. Optimized agronomic practices require mineral and/or biological fertilizer applications. The aim of this study was to determine the effects of application of a complete (N, P, and K) mineral fertilizer and vesicular arbuscular mycorrhizae (VAM) inoculation on the composition, antioxidant, and insecticidal properties of mint timija essential oils (EOs). The GC-MS analyses identified 27 components representing more than 99.9% of the total oils. Menthone (40.7–49.3%), pulegone (31.3–36.5%), and isomenthone (2.5–4.4%) were found to be the main constituents. Cultivation of mint timija with mineral fertilizer and VAM inoculation induced an increase in menthone content and a parallel decrease of pulegone. Both treatments enhanced the antioxidant activity of the investigated EOs in all assays (IC_50_ ranged from 2.34 ± 0.03 mg/mL to 6.82 ± 0.25 mg/mL), while no significant difference in the toxicities of these oils against* Tribolium confusum* du Val. has been observed. Overall, we conclude that cultivation using complete mineral fertilizer and VAM inoculation could be useful in modulating the chemical composition and enhancing the antioxidant activity of the EO of this endemic Moroccan species.

## 1. Introduction

The majority of* Mentha* species are considered as medicinal and aromatic plants and are also known to possess several biological and pharmacological properties, primarily due to their essential oils (EOs) contents [1, 2]. A total of ten* Mentha* species have been reported in the flora of Morocco [3], two of which,* M. suaveolens *subsp.* timija* and* M. gattefossei*, being endemic.* M. suaveolens* subsp.* timija* (Briq.) Harley (locally called* timija*) is a perennial herb that finds natural habitat in semiarid and subhumid areas along rivers [3]. In Moroccan folk medicine, the powder and infusion of aerial parts of this plant are also used for the treatment of whooping cough, bronchitis, and ulcerative colitis and are known for their antispasmodic and carminative properties [4]. On the other hand, it has been reported that the EOs isolated from the aerial parts of mint timija exhibit a variety of biological activities including antimicrobial [5], antioxidant, and insecticidal activities [6, 7].

Mint timija, as with other aromatic and medicinal plants, is frequently collected from its natural habitat. Unfortunately, it has been reported that overharvesting is causing a dramatic reduction in the natural populations of this endemic plant and increases its extinction risk [8]. Our previous study concluded that cultivation may constitute a rational solution to the management, conservation, and sustainable exploitation of this valuable medicinal plant [5]. However, it has been observed that this practice has increased the amount of pulegone in the EO extracted from mint timija. This compound at higher doses is known for its toxicity and its dangerous effects to human health [9]. Indeed, it has been shown that the toxicity of pulegone is mainly due to its transformation into menthofuran, considered as a violent toxin against the lungs and liver [10].

It is well known that many factors and practices related to cultivation can improve the growth and EO quality of many aromatic plants [11]. Among these practices, application of mineral and biological fertilizers, such as N, P, and K amendments and mycorrhizal inoculation have been recommended [12, 13]. In fact, the use of these cultural practices has many advantages, which include promoting the physical and biological properties of soil [14], stimulating plant growth and biomass yield through improving nutrient uptake [15], and enhancing the content and quality of EOs via modulation of the levels of enzymes that participate in the biosynthesis of volatile terpenes [16].

Several studies have shown that the application of mineral fertilizers and biological agents, such as vesicular arbuscular mycorrhizae (VAM), can influence the yield, composition, and biological properties of many medicinal and aromatic plants EOs, such as* Mentha piperita*,* Mentha viridis*,* Mentha arvensis*,* Artemisia annua*,* Rosmarinus officinalis*,* Thymus maroccanus*, and* Thymus leptobotrys *[12, 17–21]. However, no information is available on the influence of these treatments on the quality and quantity of EO in mint timija plants. Thus, the objective of the study described herein was to determine for the first time the possible effect of complete (N, P, and K) fertilizer and VAM inoculation on the chemical profile, and consequently on the insecticidal and antioxidant activities of EOs extracted from this Moroccan endemic plant.

## 2. Materials and Methods

### 2.1. Experimental Site

The experiment was carried out at the Faculty of Science Semlalia in Marrakech (31°37′N–08°02′W, 470 m above sea level). The experimental area is characterized by an arid climate with an average temperature in the warmest month of 38.3°C and of 4.5°C in the coldest one and a mean rainfall of 242 mm/year. The soil characteristics were as follows: sand (37.3%), silt (24.2%), clay (38.5%), pH (8.06), electrical conductivity (0.28 mS/cm), organic matter (2.9%), total N (0.65%), P (7.96 mg/100 g), K (40.58 mg/100 g), and Ca (18.22%).

### 2.2. Crop Experimental Design

In February 2012, a total of 400 stem cuttings of* M. suaveolens* subsp.* timija* (vegetative stage) were randomly sampled from the natural population located in the Marrakech region, Ourika valley (31°23′N–7°42′W; altitude: 900 m). The species identification was done by one of the authors (A. Abbad) and a voucher specimen (MST05) was deposited at the Laboratory of Biotechnology, Protection and Valorization of Plant Resources, Faculty of Science Semlalia, Marrakech, Morocco. The prepared cuttings (length 20 cm) were immediately transplanted to the main field in a randomized complete block design. Twenty-five cuttings were transplanted in each block (2 m × 2 m) with plant to plant and row to row spacing of 40 cm. One meter alley was maintained between treatments to eliminate any influence of lateral water movement. At the time of transplantation, the treatments were applied. Each treatment was replicated three times. Two treatments consisting of application of a complete mineral fertilizer using the N, P, and K fertilizer (60 kg/ha of N, P_2_O_5_, and K2O with 13 : 40 : 13 formulation) [21] and inoculation with 50 g/plant of* Glomus *sp. soil were conducted. Culture of* Glomus *sp. was kindly provided by Sfairi (high commissariat for water and forests and desertification in Morocco). Blocks containing the crop without any treatment were taken as control. For the first month (establishment phase), the crop was watered on alternate days. Thereafter, the irrigation was carried out as needed, depending on the rainfall. The plants were harvested at the beginning of flowering stage, 10 weeks after application of treatments. The mycorrhizal colonization of the randomized mint timija plants was determined by staining of colonized roots with trypan blue according to the method of Phillips and Hayman [22].

### 2.3. EO Extraction

For the EO extraction, we followed the methods described by Kasrati et al. [7]. The collected material was air-dried at room temperature (≈25°C) in the shade and subjected to hydrodistillation, using a Clevenger-type apparatus for 3 h until total recovery of oil. The extraction was performed on three different samples and the obtained oils were dried with anhydrous sodium sulphate, weighed, and stored at 4°C until use.

### 2.4. Gas Chromatography/Mass Spectrometry (GC/MS) Analyses of EOs

The GC/MS analysis of EOs was performed in an Agilent GC-MSD system (Agilent Technologies 6890/5973) with helium (high purity) as the carrier gas at a constant linear velocity of 37 cm/s. The transfer, source, and quadrupole temperatures were 280°C, 230°C, and 150°C, respectively, operating at 70 eV ionisation energy and scanning the *m*/*z* range 41–450. The column used was an Agilent DB5 ms capillary column (30.0 m × 0.25 mm ID × 0.25 *μ*m film thickness; model number: 122-5532) programmed from 60°C to 246°C at 3°C/min. EO samples (60 *μ*L) were diluted with acetone (2 mL). The injection volume was 1.0 *μ*L, the split ratio was 1 : 50, and the injector temperature was 260°C. Identification of the individual components was based on (i) comparison with the mass spectra of authentic reference compounds where possible and with reference to WILEY275, NBS75 K, and Adams terpene library [23] and (ii) comparison of their retention indices (RI) on a DB5 (apolar, 5% phenyl polysilphenylene-siloxane), calculated relative to the retention times of a series of C-9 to C-24* n*-alkanes, with linear interpolation, with those of authentic compounds or literature data. For semiquantitative purposes, the normalized peak area of each compound was used without any correction factors to establish abundances.

### 2.5. Antioxidant Activity

#### 2.5.1. DPPH Free Radical-Scavenging Activity

The ability of EOs to scavenge the 2,2′-diphenyl-1-picrylhydrazyl (DPPH) radical was measured using the method described by Sahin et al. [24]. The sample concentration providing 50% inhibition (IC_50_) was calculated by plotting the inhibition percentages against the concentrations of the sample.

#### 2.5.2. Reducing Power Determination

The ferric reducing capacity of EOs was determined by using the potassium ferricyanide-ferric chloride method [25]. The sample concentration providing 0.5 of absorbance (IC_50_) was calculated by plotting absorbance at 700 nm against the corresponding sample concentration.

#### 2.5.3. *β*-Carotene/Linoleic Acid Bleaching Assay

The antioxidant activity of the EOs was evaluated by the *β*-carotene/linoleic model system using the method of Miraliakbari and Shahidi [26]. The oil concentration providing 50% inhibition (IC_50_) was obtained plotting inhibition percentage versus oil solutions concentrations.

### 2.6. Insecticidal Activity 

#### 2.6.1. Insect Cultures

We followed the methods of Kasrati et al. [7]. Colonies of the red flour beetle,* Tribolium confusum* (du Val) (Coleoptera: Tenebrionidae), were maintained in the laboratory without exposure to any insecticide. They were reared in glass containers (16 cm diameter × 22 cm height) covered by a fine mesh cloth for ventilation. Each container contained a mixture of wheat flour, wheat germ, and yeast extract (13 : 6 : 1 w/w/w). The cultures were maintained in a growth chamber at 26 ± 1°C, with a relative humidity (RH) of 70–85% and 16 : 8 h light : dark photoperiod. Only young adults (7–14 days old) were used for the tests. All experimental procedures were conducted under environmental conditions identical to those of the cultures. In all bioassays, insects where considered dead when no leg or antennal movements were observed. The bioassays were designed to assess median lethal concentrations (LC_50_ and LC_90_ values) (doses that killed 50% and 90% of the exposed insects, resp.).

#### 2.6.2. Contact Toxicity Bioassay

The contact insecticidal activity of EOs obtained from mint timija against* T. confusum* adults was determined by assessing the toxicity using filter paper discs (Whatman number 1, 9 cm diameter) as described by Kasrati et al. [7]. Oils were dissolved in acetone at concentrations of 0.09, 0.16, 0.24, and 0.31 *μ*L/cm^2^. Several preliminary tests were conducted to select the doses to be used for each EO. One mL of each solution was dispensed on the surface of the filter paper that was then placed in a glass Petri dish. Control filter papers were treated with acetone only. After 10 min, once the solvent had been evaporated, 10 unsexed adults were deposited into each dish. Each EO and control treatment was replicated three times, repeating each assay twice. Mortality was recorded after 24 hours.

#### 2.6.3. Fumigant Toxicity Bioassay

The fumigant toxicity of mint timija EOs was determined using the method described by Kasrati el al. [6]. 2 cm diameter filter papers (Whatman number 1) were impregnated with the different oil doses 1, 2, 3, and 4 *μ*L. The impregnated filter papers were then attached to the screw caps of 60 mL Plexiglas bottles to give calculated fumigant concentrations of, respectively, 16.66, 33.33, 50, and 66.66 *μ*L/L air. Caps were screwed tightly on the vials, each of which contained 10 unsexed adults. Each EO and control treatment was replicated three times, repeating each assay twice. Mortality was recorded after 24 h.

### 2.7. Data Analysis

The results of antioxidant activity of EOs were expressed as the mean of three replications ± standard deviation (±SD). Variance analysis of the data (one-way ANOVA) was performed using SPSS 12.0 software and means were separated according to Student–Newman-Keuls test at *p* < 0.05. Probit analysis [27] was conducted on the corrected mortality data [28] to estimate lethal concentrations (LC_50_ and LC_90_) with their 95% confidence intervals by SPSS 12.0 Statistical Software. LC values were considered significantly different when their respective 95% confidence intervals did not overlap.

## 3. Results and Discussion

### 3.1. EO Content and Chemical Composition

The EOs from the aerial parts of* M. suaveolens* subsp.* timija* were found to be a yellow liquid with yields ranging from 0.82 ± 0.12% to 0.90 ± 0.08% (Table 1). These results demonstrated that cultivation of this species with N, P, and K fertilizer or VAM inoculation had no significant effect on the EO production. These findings are in line with what has been reported for other aromatic and medicinal species exposed to these treatments, such as* Thymus maroccanus* and* T. leptobotrys* [21],* Rosmarinus officinalis* [20], and* Lippia citriodora* [29].

Chemical analyses data of the volatile constituents of the different mint timija EOs (percentage content of each compound, elution order, retention index, and structural subclass) are summarized in Table 1. In total, 27 components were identified, which represented more than 99.9% of the total oils. Oxygenated monoterpenes (87.9–93.2%) constituted the principal compound class, followed by monoterpene hydrocarbons and sesquiterpene hydrocarbons. The predominant constituents were menthone (40.7–49.3%), followed by pulegone (31.3–36.5%), isomenthone (2.5–4.3%), 1,8-cineole (2.1–3.4%), borneol (2.3–2.7%), and* E*-caryophyllene (1.6–1.9%). The results indicate that N, P, and K fertilization and VAM inoculation affected the chemical composition of mint timija EOs, especially the content of their major compounds menthone and pulegone. In fact, compared with the oil extracted from control plants, applying both treatments produced an increase in menthone content (11.1% for N, P, and K fertilization, and 21.1% for VAM inoculation), and a reduction in pulegone (14.2% for N, P, and K fertilizer, and 11.0% for VAM inoculation).

The variation in the content of the major compounds of the EOs in response to the application of mineral and/or biological treatments observed in this study is in agreement with what has been previously reported by many authors [12, 21, 30–32]. In fact, it has been found that the addition of these treatments improved the physical and chemical properties of the soil through increases in the contents of mineral elements and organic matter and enhanced rhizosphere activity [33]. Enhancement of edaphic conditions is likely to lead to positive effects on plant growth, metabolic reactions, and enzyme activities, such as those of the terpenes biosynthesis pathways [16, 33]. In addition, many studies have shown that the application of mineral fertilizers and biological amendments plays a beneficial role in the tolerance of cultivated plants against many environmental stresses [13, 34–36]. It has been reported that cultivation of* M. suaveolens* subsp.* timija* in harsh environmental conditions such as an arid climate, low soil nutrient status, and salt stress produces an increase in pulegone content and consequent decrease in menthone content, in line with their known inverse accumulation trends [5]. Based on the results of the present study, it seems that the use of mineral fertilizer and VAM inoculation during cultivation of mint timija minimizes the effect of the environmental stress. This may indeed explain the observed increase in menthone content and decrease in pulegone.

### 3.2. Antioxidant Activity

The antioxidant capacity of mint timija EOs was assessed by three complementary* in vitro* assays. The results showed that all investigated EOs had important antioxidant properties (Table 2). The data of the analysis of variance revealed that treatments affect significantly (*p* < 0.05) the antioxidant potency of mint timija EOs. In fact, the oils obtained from the aerial parts of mint timija cultivated with mineral fertilizer displayed the highest antioxidant activities across the three assays, with IC_50_ values ranging from 2.34 ± 0.03 mg/mL to 5.96 ± 0.29 mg/mL. The lowest contribution was observed for the control plants EOs with IC_50_ values ranging from 3.29 ± 0.17 mg/mL to 7.00 ± 0.48 mg/mL. The oils obtained from plants treated with VAM inoculation expressed intermediate antioxidant potencies (IC_50_: 2.86 ± 0.04 mg/mL to 6.82 ± 0.25 mg/mL). Furthermore, for all assays, mint timija EOs were less potent than the reference antioxidants butylated hydroxytoluene (BHT) and quercetin (IC_50_ values ranged from 4.21 ± 0.08 *μ*g/mL to 7.09 ± 0.10 *μ*g/mL and from 0.95 ± 0.02 *μ*g/mL to 2.29 ± 0.10 *μ*g/mL, resp.).

The data indicate that the use of N, P, and K fertilizer and VAM inoculation had a positive influence on the antioxidant potency of* M. suaveolens *subsp.* timija* EOs. Similar results have been reported for other medicinal and aromatic plants [21, 37, 38]. The antioxidant activity of mint timija EOs is thought to correlate to its high levels of the oxygenated monoterpenes menthone and pulegone [39, 40]. However, it seems to be a general consensus that the antioxidant effects of the EOs cannot simply be explained by the action of their major components [41]. In the present study, increased activities observed for the oils obtained from mint timija cultivated with both treatments compared with the control plants only matched increases of one major compound (Table 1), so that contributions of minor compounds cannot be ruled out. Indeed, the EOs are, from the chemical point of view, quite complex mixtures; synergistic and antagonistic effects associated with minor and/or major compounds should therefore also be considered [41].

### 3.3. Insecticidal Activity

The toxicity values (LC_50_ and LC_90_) for mint timija EOs against the important stored product insect pest,* T. confusum,* using contact and fumigant methods are presented in Tables 3 and 4. Based on the calculated LC_50_ and LC_90_ values, the tested EOs exhibited potent insecticidal activity against adult* T. confusum*, confirming our previous findings [6, 7]. The LC_50_ and LC_90_ values are ranged from 0.10 to 0.13 *μ*L/cm^2^ and from 0.28 to 0.37 *μ*L/cm^2^, respectively, in contact assays as well as from 21.60 to 23.97 *μ*L/L air and from 38.35 to 54.38 *μ*L/L air, respectively, in fumigant assays. The levels of insecticidal activity of mint timija EOs registered in this work against* T. confusum *appear to be linked to the major components menthone and pulegone. Indeed, the strong toxicity of these compounds against several storage pest insects has been reported in many previous studies [2, 42, 43]. In addition, it is worth noting that the application of the treatments (N, P, and K fertilizer and VAM inoculation) has no significant effect on the insecticidal activity of mint timija EOs, since no overlap between the 95% confidence limits was observed (Tables 3 and 4). To our knowledge, there has been no previous report regarding the effect of mineral fertilizers and VAM inoculation on the insecticidal activity of plant EOs.

## 4. Conclusion

In summary, our results demonstrated that mineral fertilizer and VAM inoculation have no effect on mint timija EO yield. However, the application of both treatments induced a change in EO chemical composition with an increase of menthone content and a decrease of pulegone. Mint timija EOs subjected to both treatments presented the highest antioxidant activity, while no significant effect on the toxicity against adults of* T. confusum* has been observed. Data of this study suggest that cultivation of mint timija with N, P, and K fertilizer and VAM inoculation could be a promising solution to ensure the conservation of this valuable and endemic Moroccan species and also to enhance the chemical profile by reducing pulegone content, well known as toxic compound, and to preserve the antioxidant and insecticidal potencies of its valued EOs.

## Figures and Tables

**Table 1:** Main chemical compounds of essential oil obtained from aerial part of Moroccan *M. suaveolens* subsp. *timija* cultivated under mineral and biological fertilizers.

No	Compounds^a^	RI^b^	Untreated control	N, P, and K fertilizer	Mycorrhizal inoculation
1	*α*-Pinene	936	1.4	1.5	0.6
2	Camphene	951	1.4	1.1	0.6
3	Sabinene	975	0.7	0.9	0.4
4	*β*-Pinene	980	1.4	1.6	0.8
5	Myrcene	991	0.3	0.5	tr
6	Limonene	1030	1.2	1.2	0.7
7	*cis*-Ocimene	1033	—^c^	0.4	tr
8	1,8-Cineole	1036	2.1	3.4	2.1
9	trans-Sabinene hydrate	1068	0.3	0.3	0.4
10	Linalool	1100	0.5	0.5	0.6
*11*	*Menthone*	*1156*	*40.7*	*45.2*	*49.3*
12	Isomenthone	1166	4.3	2.5	3.4
13	Borneol	1168	2.7	2.3	2.7
14	*cis-iso*-Pulegone	1177	0.9	1.0	0.8
15	*α*-Terpineol	1192	—^c^	0.2	tr
*16*	*Pulegone*	*1242*	*36.5*	*31.3*	*32.5*
17	Piperitone	1255	0.6	0.5	0.3
18	Bornyl acetate	1288	0.5	0.3	0.5
19	Piperitenone	1342	0.3	0.3	0.4
20	Piperitenone oxide	1367	0.3	0.2	0.3
21	*E*-Caryophyllene	1425	1.8	1.9	1.6
22	*α*-Humulene	1459	—^c^	0.3	tr
23	Germacrene D	1486	1.1	1.9	1.4
24	Bicyclogermacrene	1502	—^c^	0.4	tr
25	Spathulenol	1534	0.3	—^c^	—^c^
26	Caryophyllene oxide	1589	0.8	0.2	0.8
27	*α*-Cadinol	1645	—^c^	0.2	tr
	*Monoterpene hydrocarbons *		*6.5*	*7.2*	*3.0*
	*Oxygenated monoterpenes *		*89.6*	*87.9*	*93.2*
	*Sesquiterpene hydrocarbons *		*2.9*	*4.4*	*3.0*
	*Oxygenated sesquiterpenes *		*1.0*	*0.4*	*0.8*
	*Total (%)*		*100*	*99.9*	*100*
	Yields (%)		0.90 ± 0.08	0.84 ± 0.10	0.82 ± 0.12

^a^Compounds listed in order of elution. ^b^RI (retention indices) measured relative to *n*-alkanes (C-9 to C-24) on the nonpolar DB-5 column. tr: traces (<0.1%). ^c^Not detected.

**Table 2:** IC_50_ values of mint timija essential oils, BHT, and quercetin.

Antioxidant tests	Essential oils (mg/mL)			Standard antioxidants (*µ*g/mL)	
	Untreated control	N, P, and K fertilizer	Mycorrhizal inoculation	Quercetin	BHT
DPPH	3.29 ± 0.17^a^	2.34 ± 0.03^c^	2.86 ± 0.04^b^	1.07 ± 0.01^d^	4.21 ± 0.08^d^
Reducing power	7.00 ± 0.48^a^	5.96 ± 0.29^c^	6.82 ± 0.25^b^	2.29 ± 0.10^d^	7.09 ± 0.10^d^
*β*-Carotene/linoleic acid	4.06 ± 0.30^a^	3.23 ± 0.14^c^	3.50 ± 0.13^b^	0.95 ± 0.02^d^	4.30 ± 0.33^d^

Values are given as mean ± SD (*n* = 3). Values with different letters under the same line are significantly different (*p* > 0.05).

**Table 3:** Lethal concentrations (LC_50_ and LC_90_) values of mint timija essential oils applied by contact toxicity bioassay against *T. confusum.*

Essential oils	LC_50_ (*µ*L/cm^2^)	LC_90_ (*µ*L/cm^2^)	Slope ± SE	*χ* ^2^	Df^b^
	(95% CL)^a^	(95% CL)			
Untreated control	0.13 (0.06–0.18)	0.37 (0.22–1.19)	3.24 ± 1.12	0.59	2
N, P, and K fertilizer	0.10 (0.02–0.15)	0.28 (0.19–1.08)	2.99 ± 1.15	0.63	2
Mycorrhizal inoculation	0.12 (0.05–0.20)	0.33 (0.28–1.83)	4.32 ± 1.29	0.28	2

^a^95% lower and upper confidence limits are shown in parenthesis. ^b^Degree of freedom; SE: standard error. LC values are considered significantly different when 95% CL did not overlap.

**Table 4:** Lethal concentrations (LC_50_ and LC_90_) values of mint timija essential oils applied by fumigant toxicity bioassay against *T. confusum*.

Essential oils	LC_50_ (*µ*L/L air)	LC_90_ (*µ*L/L air)	Slope ± SE	*χ*^2^	Df^b^
	(95% CL)^a^	(95% CL)			
Untreated control	23.97 (15.23–31.19)	54.38 (35.53–90.83)	4.39 ± 1.22	0.40	2
N, P, and K fertilizer	21.60 (15.16–27.51)	38.35 (27.71–63.97)	5.92 ± 1.78	0.30	2
Mycorrhizal inoculation	23.14 (11.52–31.52)	46.93 (41.42–99.03)	3.45 ± 1.09	1.15	2

^a^95% lower and upper confidence limits are shown in parenthesis. ^b^Degree of freedom; SE: standard error. LC values are considered significantly different when 95% CL did not overlap.
